# Intrinsic laws of k-mer spectra of genome sequences and evolution mechanism of genomes

**DOI:** 10.1186/s12862-020-01723-3

**Published:** 2020-11-23

**Authors:** Zhenhua Yang, Hong Li, Yun Jia, Yan Zheng, Hu Meng, Tonglaga Bao, Xiaolong Li, Liaofu Luo

**Affiliations:** 1 Laboratory of Theoretical Biophysics, School of Physical Science & Technology, Inner Mongolia University, Hohhot, 010021 China; 2 School of Economics and Management, Inner Mongolia University of Science & Technology, Baotou, 014010 China; 3 College of Science, Inner Mongolia University of Technology, Hohhot, 010051 China; 4 Baotou Medical College, Inner Mongolia University of Science & Technology, Baotou, 014040 China; 5 School of Life Science & Technology, Inner Mongolia University of Science & Technology, Baotou, 014010 China

**Keywords:** Genome sequence, K-mer spectra, Independent selection law, Evolution mechanism of genomes, Evolution modes of prokaryotes

## Abstract

**Background:**

K-mer spectra of DNA sequences contain important information about sequence composition and sequence evolution. We want to reveal the evolution rules of genome sequences by studying the k-mer spectra of genome sequences.

**Results:**

The intrinsic laws of k-mer spectra of 920 genome sequences from primate to prokaryote were analyzed. We found that there are two types of evolution selection modes in genome sequences, named as CG Independent Selection and TA Independent Selection. There is a mutual inhibition relationship between CG and TA independent selections. We found that the intensity of CG and TA independent selections correlates closely with genome evolution and G + C content of genome sequences. The living habits of species are related closely to the independent selection modes adopted by species genomes. Consequently, we proposed an evolution mechanism of genomes in which the genome evolution is determined by the intensities of the CG and TA independent selections and the mutual inhibition relationship. Besides, by the evolution mechanism of genomes, we speculated the evolution modes of prokaryotes in mild and extreme environments in the anaerobic age and the evolving process of prokaryotes from anaerobic to aerobic environment on earth as well as the originations of different eukaryotes.

**Conclusion:**

We found that there are two independent selection modes in genome sequences. The evolution of genome sequence is determined by the two independent selection modes and the mutual inhibition relationship between them.

## Background

The frequency of k-mers (k = 1, 2, 3…) in nucleotide sequences is nonrandom. The nonrandom characteristic was widely used to predict and identify functional regions, such as promoter regions [1–3, 5], enhancers [4], CpG island sequences [5, 6], conservative non-coding sequences [7] and transcriptional start sites [8]. The motif characters of k-mers have been used to analyze the interaction signals between nucleotide elements and proteins, such as recognizing the hypersensitive binding site of enzymes [9], probe design [10], drug design [11] and nucleosome positioning [12, 13]. The usage difference of k-mers has been used to do the sequence alignment [14] in chromosome assemble [15, 16], genome dictionary construction [17, 18] and metagenomic classification [19, 20], etc. Although advances have been made in information mining of nucleotide sequences, the composition rules of nucleotide sequences remain unclear.

The proprietary characteristics of nucleotide sequences were also used to describe the species evolution relationships. The valuable achievement is Carl Woese’s work [21]. They used the conserved (SSU) rRNA sequence to construct phylogenetic trees and proposed the Three Domain theory [22]. Later, the single conserved protein-coding gene has been used to build phylogenetic trees, such as EF-Tu gene [23], Hsp60 gene [24], the largest subunit of RNA polymerase [25] and aminoacyl tRNA enzyme gene [26], etc. In order to improve the accuracy and consistency of phylogenetic relationship, the conserved gene set has been used to replace single gene [27–29]. Lang and Eisen used 24 of the most conserved genes [30], Ciccarelli et al. used 31 tandem genes [31] and Hao Bolin used all the protein sequences of prokaryote genome [32, 33]. Some people tried to use the total k-mer frequencies of genome sequences to construct phylogenetic trees, but the results were not satisfactory. In order to get the consistency with the accepted phylogenetic tree, the total k-mer set had to be screened [34–38]. It is known that the perfect idea is to use the information of whole-genome sequence to characterize genome evolution. It is clearly inappropriate to use both the conserved gene set and part of k-mers to characterize genome evolution, and it is hard to determine the complete and consistent set of the k-mers or conserved genes. The study of phylogenetic relationship in genome level has encountered a bottleneck.

Some researchers focused on the k-mer spectral distributions of genome sequences. Chen first analyzed the k-mer (k = 6) spectra of 9 genome sequences [39]. Beny Chor then studied the k-mer (k = 7–11) spectra of nearly 100 genome sequences [40]. They found that the k-mer spectra are unimodal in a few of the other vertebrates, fungi and prokaryotes and tri-modal in four-clawed mammals. Benny Chor believed that the differences of k-mer spectra are caused by the interaction between the CG dinucleotide ratio and the G + C content. Our previous study showed that the k-mer spectrum distributions of genome sequences, as a window, revealed the laws of the composition and evolution of genome sequences [41, 42]. We found that the 8-mers spectrum distributions correlate with genome evolution [43] and the 8-mers containing CG dinucleotide are functional motifs [44, 45]. Here we studied the spectrum distributions of various k-mer subsets based on 920 genome sequences from primates to bacteria. We wish to reveal the intrinsic laws of k-mer spectrum of genome sequences and the evolution mechanism of genomes.

## Results

### K-mer spectra of genome sequences

In order to choose the proper *k* value to construct the reliable k-mer spectrum, the k-mer spectrum of human genome sequence was obtained with *k* value from 6 to 13. We found that when *k* ≥ 6, the k-mer spectrum distribution tends to be stable gradually. According to the statistical theory, the chosen *k* value must ensure that the frequency of k-mer with the lowest frequency must be guaranteed to meet the statistical significance in a given DNA sequence [46, 47]. Beny Chor proposed a formula *k* = 0.7*log*_4_ *L* to estimate the minimum *k* value, *L* is the length of the given DNA sequence [40]. In eukaryote genomes, the yeast genome is short and the calculated *k* value is 8.9. In analyzed prokaryote genomes, the calculated *k* value is larger than 6. Without loss of generality, 8-mer was selected in eukaryotic genomes and 6-mer was selected in prokaryotic genomes in our analysis. For the convenience of statement, except for special cases, the 8-mer and the 6-mer are uniformly expressed as the motif.

**Fig. 1:**
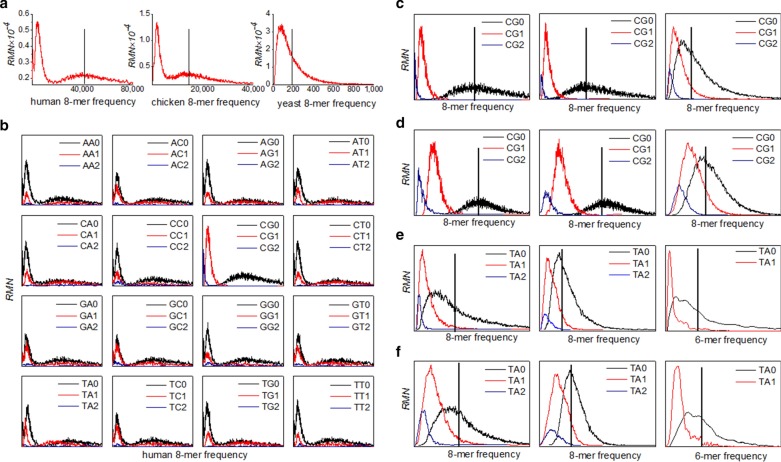
Motif spectrum distributions of genome sequences. **a** Motif spectra of three representative genome sequences. Of human genome is shown to represent the tri-modal spectrum, of chicken genome is shown to represent quasi-di-modal and of yeast genome is shown to represent unimodal. The vertical bar is the center frequency of the corresponding random sequence. **b** The spectra of XY2, XY1 and XY0 motif subsets of human genome sequence (X, Y = A, C, G, T). **c** The spectra of CG2, CG1 and CG0 motif subsets in human, chicken and yeast genome sequences. **d** The normalized spectra of CG2, CG1 and CG0 motif subsets in which the motif frequencies are transformed by the square root transformation in human, chicken and yeast genome sequences. **e** The spectra of TA2, TA1 and TA0 motif subsets of three representative genome sequences. *Tetrabaena socialis* is green algae (left), *Schizophyllum commune* is fungus (middle) and *Halomicrobium katesii* is prokaryote (right). **f** The normalized spectra of TA2, TA1 and TA0 motif subsets in which the k-mer frequencies are transformed by the square root transformation in the three representative genome sequences

The motif spectra of 920 genome sequences (Table 1 and Additional file 1: Table S1) were obtained. For animal genomes, the motif spectrum distributions are tri-modal in mammal genomes, unimodal in invertebrate and quasi-di-modal in other vertebrate genome sequences. It is consistent with the previous conclusions [39, 40, 45]. But the motif spectra in plant, fungi and bacteria genomes are a little more complicated. The motif spectra are unimodal for most of the genomes and di-modal or quasi-di-modal for few of the genomes. Here, only the motif spectra of three representative genome sequences were shown in Fig. 1a, which stand for the tri-modal, quasi-di-modal and unimodal spectra respectively. With the genome evolution from fungi, invertebrates to primates, we can see that the motif spectrum of genome sequence transfers gradually from unimodal to tri-modal. It indicates that the motif spectrum of genome sequence is closely related to genome evolution.

**Table 1:** Genome data and its classification

Group	Abbr	No	Group	Abbr	No	Group	Abbr	No
Vertebrate		*69*	*Plant*		*63*	Thermococci	Tpr	33
Primates	Pri	13	Dicotyledons	Dic	28	Thermoprotei	Tco	29
Rodents	Rod	14	Monocotyledons	Mon	11	Methanomada	Mma	50
Other mammals	Mam	22	Pteridophyta	Pte	3	*Eubacteria*		*300*
Other vertebrates	Vrt	20	Green algae	Gal	21	Actinobacteria	Act	67
Invertebrate		*43*	*Fungus*		*245*	Bacteroidetes	Bac	44
Coleoptera	Col	3	Agaricomycotina	Aga	73	Cyanobacteria	Cya	41
Diptera	Dip	21	Pezizomycotina	Pez	118	Firmicutes	Fir	50
Hymenoptera	Hym	6	Saccharomycetales	Sac	54	Proteobacteria	Pro	53
Lepidoptera	Lep	4	*Archaea:*		*200*	Spirochaetales	Spi	45
Mollusca	Mol	3	Halobacteria	Hal	62			
Nematoda	Nem	6	Methanomicrobia	Mmi	26

In tri-modal motif spectra, the three peak distributions were called peak2, peak1 and peak0 successively from low frequency to high frequency. We found that the most probable frequency in peak2 and peak1 distributions is far below than the center frequency of random sequences. The most probable frequency in peak0 distribution is around the center frequency of random sequences. In unimodal motif spectra, the most probable frequency is lower than the center frequency of random sequences (Fig. 1a).

Why does it appear the multi-modal spectrum in genome sequences? According to statistical theory, we thought that the total motifs in tri-modal spectrum come from three different populations or genome sequences are composed of three different motifs. Do the total motifs in unimodal spectrum still come from three different populations? Only when the three kinds of motifs are separated strictly can the mechanism be revealed.

### Spectrum characteristics of motif subsets

In order to separate clearly the three motif subsets from the spectrum of tri-modal distribution, the total motifs were divided into XY0, XY1 and XY2 motif subsets (X,Y = A, C, G, T) according to the XY dinucleotide classification method ("Methods” section) and the spectra of these motif subsets were obtained in human, chicken and yeast genomes.

We found that only the spectra of CG0, CG1 and CG2 motif subsets turn up the independent unimodal distributions respectively (Fig. 1b) and the three spectra of CG2, CG1 and CG0 motif subsets corresponds strictly to the peak 2, peak 1 and peak 0 distribution of the total motif spectrum respectively in human genome sequence. Thus, the total motifs located in the tri-modal spectrum are cleanly separated. The other 15 kinds of spectra of XY0, XY1 and XY2 motif subsets have not the distribution features and their spectra are still the tri-modal distributions which are similar to the spectrum of total motifs. For the other two representative genomes, the distribution features of CG0, CG1 and CG2 motif subsets are the same as that in human genome sequence, even though their spectra of total motifs are not tri-modal (Fig. 1c). The multi-modal motif spectrum of genomes can be explained by the spectrum distributions of CG2, CG1 and CG0 motif subsets. If the distance among the spectrum of three subsets is far apart, the spectrum of total motifs superimposed by them is tri-modal. If the distance among the spectrum of three subsets is very close, the spectrum of total motifs superimposed by them is unimodal.

### CG independent selection law of genome sequence

The spectra of total motifs and the 16 kinds of XY0, XY1 and XY2 motif subsets for animal genomes were analyzed. For 49 mammal genomes with obvious tri-modal motif spectra, we found that the spectrum features of XY0, XY1 and XY2 motif subsets are the same as that in human genome sequence and the total motifs are strictly separated into three independent subsets: CG0, CG1 and CG2 motif subsets (Fig. 1c). We named this distribution property as Evolution Independence of genome sequences. Compared with the center frequency of the corresponding random sequences, we found that the frequency distribution of CG0 motif subset is around the random center, and the most probable frequency in CG1 and CG2 motif spectra is lower than that of the random center. It indicates that the occurrence frequencies of CG0 motifs are the result of random selection and the occurrence frequencies of CG1 and CG2 motifs are the result of directional selection. We named this distribution property as Evolution Selectivity of genome sequences. Besides, the spectrum distributions of CG1 and CG2 motif subsets are much narrower than that of CG0 motif subset (Fig. 1c). It means that the occurrence frequencies of CG1 and CG2 motifs are more conservative than that of CG0 motifs. We named this distribution property as Evolution Conservatism of genome sequences. Generally, the k-mers with the properties of directional selection and conservative usage were considered as functional motifs. We found that only the spectral distributions of the three kinds of CG motif subsets have the three properties, the other 15 kinds of spectra of XY2, XY1 and XY0 motif subsets do not satisfy simultaneously the three properties in mammal genomes. We named this phenomenon as CG Independent Selection Law of genome sequences and it is abbreviated as CG independent selection law. For 63 other vertebrate and invertebrate genomes, though their motif spectra are quasi-di-modal or unimodal, we found that the spectrum distributions of CG0, CG1 and CG2 motif subsets also abide by the CG independent selection law (Fig. 1c).

### TA independent selection law of genome sequences

For plant, fungi and bacteria genomes, the CG independent selection law is obvious in some species genomes, but not obvious in some other species genomes. By observing the spectrum characteristics of XY2, XY1 and XY0 motif subsets in these genomes, we found that there is another type of independent selection law. The spectrum characteristics of TA2, TA1 and TA0 motif subsets still follow the properties: the evolution independence, the evolution selectivity and the evolution conservatism (Fig. 1e). We named it as TA Independent Selection Law. The other 14 kinds of spectra of XY2, XY1 and XY0 motif subsets (besides three CG motif subsets) do not satisfy the three properties simultaneously.

Based on the above results, we re-examined the spectrum distributions of all motif subsets in all genomes from human to bacteria. We found that both CG independent selection law and TA independent selection law exist simultaneously in genomes. In general, CG independent selection law is usually obvious in some eukaryote genomes, such as in vertebrates, dicotyledons and monocotyledon and TA independent selection law is obvious in green algae.

### Quantitative characterization of CG and TA independent selections

In order to study the phenomena of the two independent selections, the quantitative characterization was given about the spectrum characteristics. For most of the species genomes, their spectra of total motifs and the XY motif subsets are unimodal. We found that these unimodal distributions are not the normal distribution, they are similar to the *χ*^2^ distribution with small degrees of freedom and odd to the left. Of this kind of distributions, its average frequency is correlated with its standard deviation. In order to use the average frequency and the standard deviation to describe the location and the degree of variation of the spectra independently, the actual distributions should be converted as close as possible to normal distributions. After the attempts, all of the motif frequencies were transformed by the square root transformation. We found that the transformed distribution is very close to the normal distribution (Fig. 1d, f).

Based on the average frequency and the standard deviation of total motif spectra and XY motif spectra, the separability and the conservatism were used to characterize the spectrum distribution (“Methods” section). For a given spectrum, its separability value is denoted as ${\delta }_{XYi}$ and its conservatism value is denoted as ${\rho }_{XYi}$ (X, Y = A, C, G, T and *i* = 0, 1, 2). The effects of motif absolute frequency and the genome scale are eliminated in the two parameters. So, the two parameters can be used to compare the difference among different spectra in a genome and among genomes. For the spectra of CG0, CG1 and CG2 motif subsets of a genome, their separability values are denoted as ${\delta }_{CG0}$, ${\delta }_{CG1}$, ${\delta }_{CG2}$ and their conservatism values are denoted as ${\rho }_{CG0}$, ${\rho }_{CG1}$, ${\rho }_{CG2}$ respectively. For the spectra of TA0, TA1 and TA2 motif subsets of a genome, their separability values are denoted as ${\delta }_{TA0}$, ${\delta }_{TA1}$, ${\delta }_{TA2}$ and their conservatism values are denoted as ${\rho }_{TA0}$, ${\rho }_{TA1}$, ${\rho }_{TA2}$ respectively. The values of these parameters for 920 genomes were showed in Additional file 1: Table S1.

### Relations between the separability and the conservatism

We found that the distributions of the separability and the conservatism for CG1 and CG2 spectra and for TA1 and TA2 spectra are similar. So, the linear correlation analysis was done between the separability and the conservatism in each species group. Results showed that the separability of CG1 and CG2 motif spectra and of TA1 and TA2 motif spectra correlates significantly and positively with the conservatism of them (Table 2). It is to say that the higher the separability is, the more conservative will be for the spectra of the motifs containing CG and TA dinucleotides. We named the distribution property as Evolution Correlation of genome sequences. It is the fourth property of CG and TA independent selection laws. Furthermore, we found that the separability of CG1 motif spectra correlates significantly and positively with that of CG2 motif spectra, the conservatism of CG1 motif spectra correlates significantly and positively with that of CG2 motif spectra. The same conclusions also happen between TA1 and TA2 motif spectra (Table 2). It indicates that CG1 and CG2 motifs subsets as well as TA1 and TA2 motifs subsets abide by the same kind of evolutionary selection pattern. We named the distribution property as Evolution Homoplasy of genome sequences. It is the fifth property of CG and TA independent selection laws. For different species groups, there are not consistent correlation patterns between the separability and conservatism of CG0/TA0 motif spectra. Some of them are positive and some of them are negative. We thought that CG0 and TA0 motifs are the fundamental ‘materials’ and reflect the basic structures of a genome sequence.

**Table 2:** Linear correlation coefficients between the separateness and conservatism of three CG 8-mer spectra and the three TA 8-mer spectra

	Pri	Rod	Mam	Vrt	Inv	Dic	Mon	Gal	Sac	Aga	Pez	Arc	Eub
	(13)	(14)	(22)	(20)	(43)	(28)	(11)	(21)	(54)	(73)	(118)	(200)	(300)
δ_CG0_-ρ_CG0_	− 0.619*	− 0.726**	− 0.612**	− 0.885**	− 0.505**	− 0.330	− 0.105	0.745**	− 0.287*	0.119	0.406**	0.865**	0.863**
δ_CG1_-δ_CG2_	0.921**	0.660*	0.928**	0.977**	0.968**	0.938**	0.801**	0.982**	0.981**	0.920**	0.871**		
δ_CG1_-ρ_CG1_	0.801**	0.736**	0.911**	0.960**	0.909**	0.872**	0.856**	0.893**	0.975**	0.925**	0.895**	0.948**	0.929**
δ_CG2_-ρ_CG2_	0.941**	0.899**	0.929**	0.850^**^	0.846**	0.769**	0.983**	0.974**	0.928**	0.863**	0.730**		
ρ_CG1_-ρ_CG2_	0.902**	0.865**	0.947**	0.887**	0.881**	0.801**	0.746**	0.929**	0.985**	0.952^*^*	0.952^**^		
δ_TA0_-ρ_TA0_	− 0.210	0.637^*^	0.233	0.165	0.793**	0.675**	0.622*	− 0.150	0.387**	− 0.765**	0.211*	0.569**	0.284**
δ_TA1_-δ_TA2_	0.157	0.708**	0.830**	0.968**	0.913^**^	0.882**	0.897**	0.976**	0.977**	0.961**	0.701**		
δ_TA1_-ρ_TA1_	− 0.183	0.457	0.180	0.321	0.546**	0.475^*^	0.753**	0.958**	0.861**	0.788**	0.876**	0.950**	0.849**
δ_TA2_-ρ_TA2_	0.464	0.657*	0.081	0.141	0.736**	0.459*	0.877**	0.955**	0.766**	0.753**	0.865**		
ρ_TA1_-ρ_TA2_	0.370	0.815**	0.452*	0.486*	0.726**	0.814**	0.913**	0.948**	0.857**	0.914**	0.668**		

Two-tailde significance:* p < 0.05;** p < 0.01

It is found that there are exceptions in vertebrate animals. In primate genomes, both the evolution correlation and the evolution homoplasy for TA1 and TA2 motif spectra have disappeared. That means the TA independent selection has disappeared in primate genomes. In rodent, other mammal and other vertebrate genomes, the evolution correlation for TA1 and TA2 motif spectra has disappeared, but the evolution homoplasy for TA1 and TA2 motif spectra still exist (Table 2). With the levels of genome evolution increasing in animals, our results show that what disappeared first is the evolution correlation, and then the evolution homoplasy.

In a word, we found out the CG and TA independent selection laws in genome sequences by analyzing the intrinsic laws of k-mer spectra of genome sequences. The CG and TA independent selection laws have five properties: evolution independence, evolution selectivity, evolution conservatism, evolution correlation and evolution homoplasy.

### Statistical test of CG and TA independent selection laws

In order to inspect the sensibility of the CG and TA independent selections in different genomes. Considering that there are positive linear correlations between the separability and the conservatism of CG1, CG2, TA1, TA2 spectra. Only the parameter of separability was calculated for the 16 kinds of XY0, XY1 motif spectra of 920 genomes and for the 16 kinds of XY2 motif spectra of 420 genomes (XY2 6-mer subset is not defined in 500 prokaryote genomes). Their variances were calculated for each genome group.

**Fig. 2:**
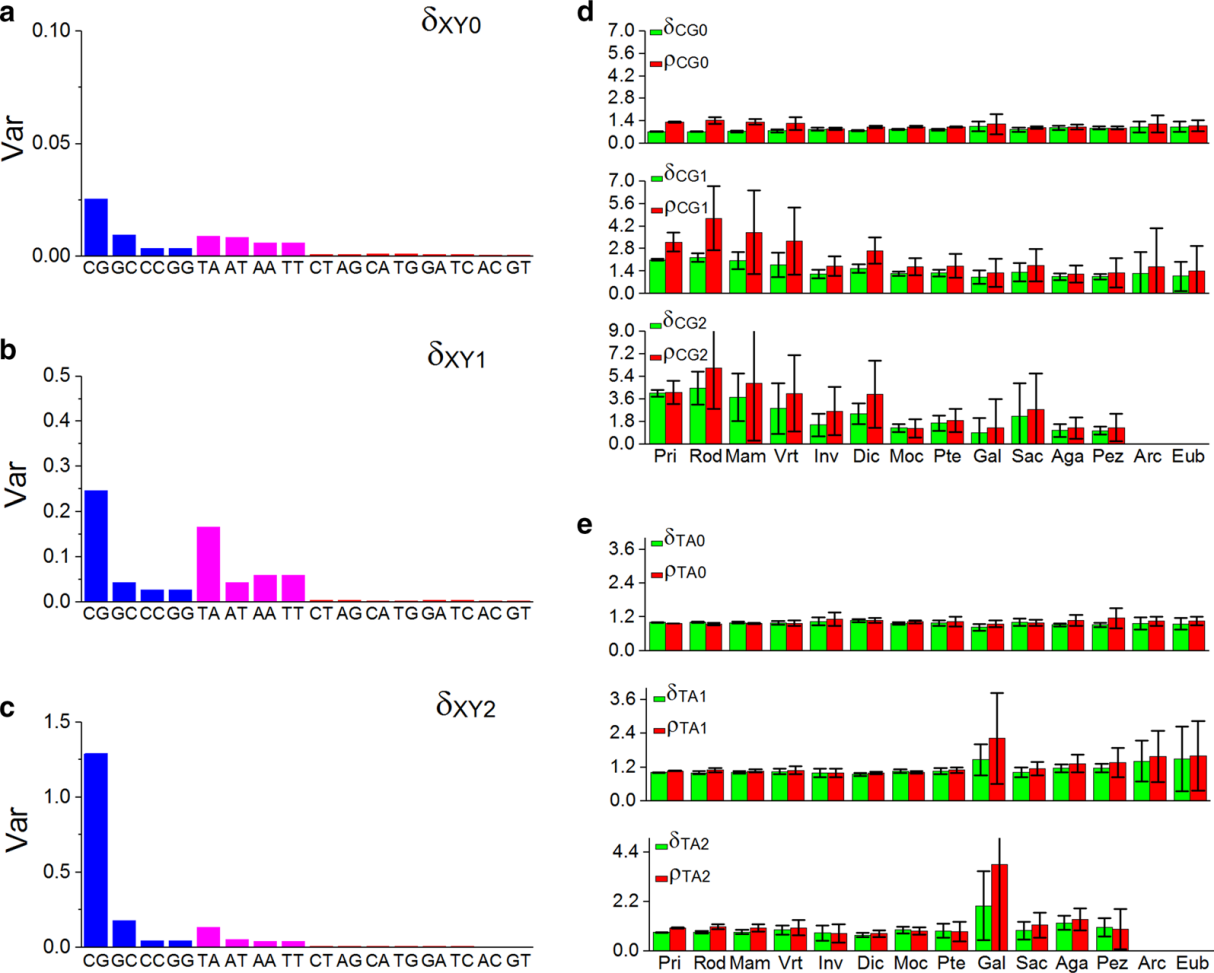
Distributions of the separability and conservatism values of XY motif spectra. **a** Variance distribution of the separability of 16 kinds of XY0 motif spectra for 920 genomes. **b** Variance distribution of the separability of 16 kinds of XY1 motif spectra for 920 genomes. **c** Variance distribution of the separability of 16 kinds of XY2 motif spectra for 420 genomes. **d** Average values and point estimation values (*p* < 0.05) of the separability and conservatism of CG0, CG1 and CG2 motif spectra in each species group. **e** Average values and point estimation values (*p* < 0.05) of the separability and the conservatism of TA0, TA1 and TA2 motif spectra in each species group

According to the distributions of the variances, we found that CG, GC, CC, GG and TA, AT, AA, TT motif sets which correlate with genome divergence, these 8 motif sets are called the correlated motif sets. The other 8 kinds of XY motif sets do not correlate with the genome divergence, those 8 motif sets are called non-correlated motif sets. When compared with XY1 and XY2 motif sets in the 8 correlated motif sets, the variances of XY0 motif sets are very small (Fig. 2a). It indicated that the characteristics of XY0 motif sets, especially CG0 and TA0 motif sets, are not sensitive to reflect the genome divergence.

For the 8 correlated motif sets of XY1 or XY2, the variance of the CG motif set in CG, GC, CC, GG motif group and of the TA motif set in TA, AT, AA, TT motif group is larger than the other three motif sets respectively (Fig. 2b, c). The homogeneity of variance test (*F-*test) was done between CG and GC/CC/GG motif spectra as well as between TA and AT/AA/TT motif spectra. Results indicated that CG1/CG2 motif sets and TA1/TA2 motif sets have significant variance heterogeneity (p < 0.01). Considering that only CG and TA motif spectra abide by the five properties of the independent selection simultaneously, we can conclude that genome evolution pressure is mainly reflected in the characteristics of CG and TA motif sets.

We found that the characteristics of GC/CC/GG and AT/AA/TT motif spectra do not abide by the five properties of the independent selection simultaneously. We considered that the characteristics of GC/CC/GG motif sets and of AT/AA/TT motif sets are the synergistic effect under the affection of CG and TA independent selection respectively. This phenomenon is worthy of further study.

### Independent selection laws and genome evolution

According to the species taxonomy (Table 1 and Additional file 1: Table S1), the point estimation values (*p* < 0.05) of the separability and the conservatism were calculated in each species group (Additional file 2: Table S2). Results showed that the average values of ${\delta }_{CG1}$/${\delta }_{CG2}$ and ${\rho }_{CG1}$/${\rho }_{CG2}$ have obvious difference among different species groups and their confidence intervals are large. The separability and conservatism of CG1 and CG2 motif spectra correlate positively with the levels of genome evolution and are very sensitive to the genomes within species groups (Fig. 2d). The average values of ${\delta }_{CG0}$ and ${\rho }_{CG0}$ change little among different species groups and their confidence intervals are relatively small. That means the separability and the conservatism of CG0 motif spectra are not sensitive to the genome evolution and the genomes within species groups relatively. Similar, the average values of ${\delta }_{TA0}$ and ${\rho }_{TA0}$ also change very little among species groups and their confidence intervals are relatively small. That means the separability and conservatism of TA0 motif spectra are not sensitive to the genome evolution and the genomes within the species groups. The average values of ${\delta }_{TA1}$/${\delta }_{TA2}$ and ${\rho }_{TA1}$/${\rho }_{TA2}$ have obvious difference among species groups and their confidence intervals are relatively large. The separability and conservatism of TA1 and TA2 motif spectra correlate negatively with the levels of genome evolution and are very sensitive to the genomes within the species groups, especially in green algae group (Fig. 2e).

### Intensity distributions of CG and TA independent selections

**Fig. 3:**
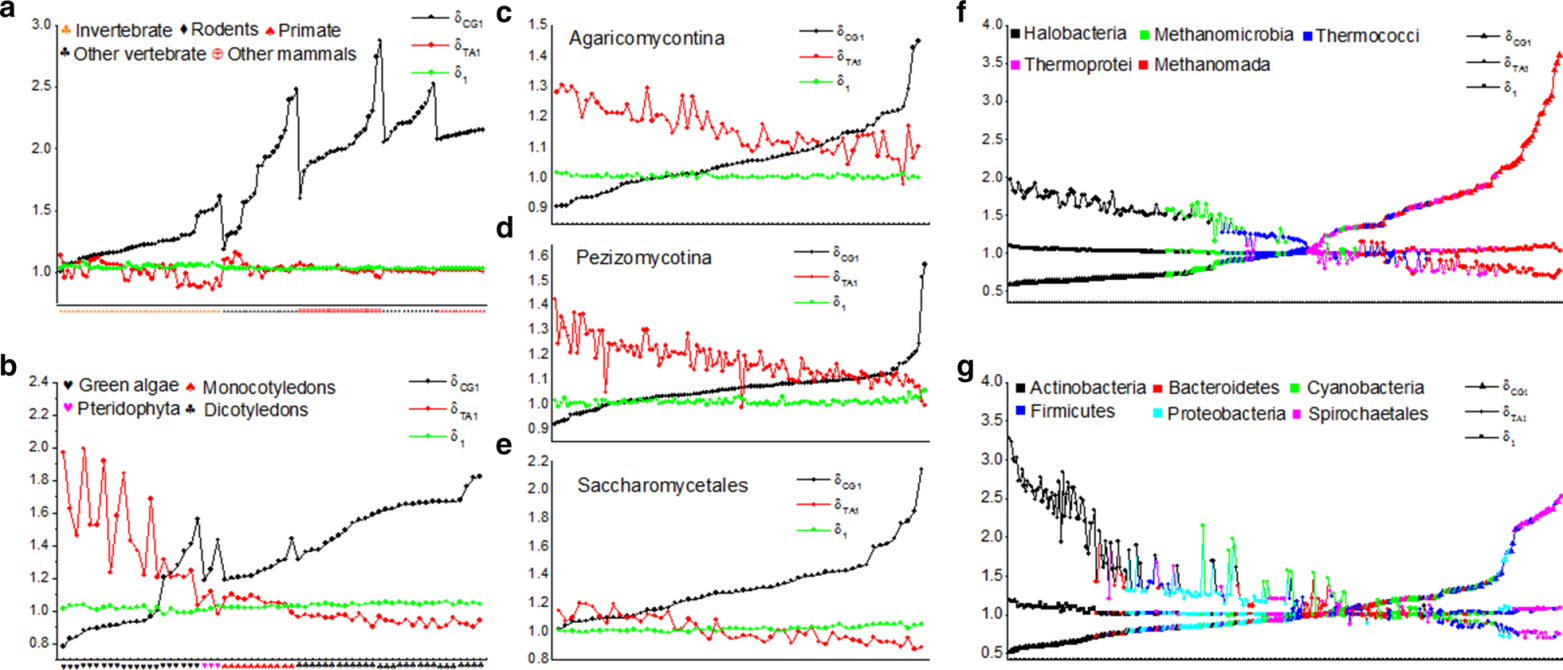
Intensity distributions of CG independent selection and TA independent selection. The separability values of CG1 and TA1 spectra represent intensity distributions of CG and TA independent selections respectively. The ${\delta }_{1}$ is the background value. The abscissa represents species genome of species groups. From left to right, the species in each species group is arranged by its separability value of CG1 motif spectrum from small to large. **a** 112 animal genomes. The abscissa is species genome which is arranged by species groups. From left to right, the order is invertebrates, other vertebrates, other mammals, rodents and primates. **b** 63 plant genomes. The abscissa is species genome which is arranged by species groups. From left to right, the order is green algae, pteridophyte, monocotyledon and dicotyledon. **c**)73 *Agaricomycotina* genomes of fungi. **d** 118 *Pezizomycotina* genomes of fungi. **e** 54 *Saccharomycetales* genomes of fungi. **f** 200 archaea genomes. The archaea genomes are classified into five subgroups: *Halobacteria* (black), *Methanomicrobia* (green), *Thermococci* (blue), *Thermoprotei* (pink), and *Methanomada* (red). **g** 300 Eubacteria genomes. The Eubacteria genomes are classified into six subgroups: *Actinobacteria* (black), *Proteobacteria* (wathet blue), *Cyanobacteria* (green), *Bacteroidetes* (red), *Firmicutes* (blue) and *Spirochaetales* (pink)

The above analysis indicated that there is a significant linear positive relationship between the separability and the conservatism of CG1, CG2, TA1 and TA2 spectra. Therefore, we only chose the separability value of CG1 spectrum (${\delta }_{CG1}$) to represent the intensity of CG independent selection and chose the separability value of TA1 spectrum (${\delta }_{TA1}$) to represent the intensity of TA independent selection for each genome. For the convenience of comparison, the average value of the separability (${\delta }_{1}$) of the other 14 XY1 motif spectra is taken as a criterion to represent the background value in each genome. If ${\delta }_{CG1}>{\delta }_{1}$ or ${\delta }_{TA1}>{\delta }_{1}$, the CG independent selection or the TA independent selection is considered to be obvious. In the following distribution figures (Fig. 3), the genomes in the abscissa are arranged in order of ${\delta }_{CG1}$ values from small to large, the names and sort orders of the genomes in each figure are shown in Additional file 1: Table S1.

#### Animal genomes

Generally, the CG independent selection is much obvious and the TA independent selection is weak in animal genomes. We found that the intensity distribution of CG independent selection presents a positive relationship with the levels of genome evolution. In some genome groups, such as vertebrate genomes, the intensity values of CG independent selection change greatly. We considered that the intensity of CG independent selection can also reflect the evolution rate of a species genome. If the intensity value is higher than its average value of a species group, the genome is evolving fast. On the contrary, if the intensity value is lower than its average value of a species group, the genome is evolving slowly. For instance, lamprey (the leftmost data of the other vertebrate group in Fig. 3a) has the smallest intensity of CG independent selection. It indicates that lamprey genome is evolving very slowly. The result is consistent with the conclusion of biologists. Of medium ground finch, zebra finch and budgerigar in other vertebrate genomes, opossum and wallaby in other mammal genomes, Chinese hamster and squirrel in rodent genomes (data in the right end of the corresponding species groups in Fig. 3a), these species have obviously higher intensity values. We considered that these organisms are evolving fast in their corresponding species groups.

The intensity values of TA independent selection fluctuate among the background ${\delta }_{1}$ values in other vertebrate group and basically disappear in other mammal, rodent and primate groups. It indicates that the TA independent selection is disappearing with the increasing of the genome evolution levels in vertebrate genomes. The results are consistent with the analysis in Table 2. In invertebrate group, we found that the intensity distribution of CG independent selection presents a negative correlation with that of the TA independent selection. The TA independent selection is inhibited by the CG independent selection. We named this phenomenon as TA Inhibition. Meanwhile, we found that the intensity values of TA independent selection are even lower obviously than the background value ${\delta }_{1}$ while the intensity values of CG independent selection are obviously high in some invertebrate genomes. It means the TA independent selection was inhibited strongly by the CG independent selections. We named this phenomenon as Strong TA Inhibition.

#### Plant genomes

We found that the negative correlation between CG and TA independent selections is obvious and not only the TA independent selection is inhibited by the CG independent selection but also the CG independent selection is inhibited by the TA independent selection. Combining the results in invertebrate genomes, we concluded that the inhibition phenomenon is mutual. We named the phenomenon as Mutual Inhibition (Additional file 3: Animation S1). When comparing the distribution modes of some green algae genomes with that of dicotyledon genomes (Fig. 3b), we found that the phenomenon of the strong mutual inhibition also exists in plant genomes.

According to the general consensus of biologists, the order of species evolution level is green algae, pteridophyte, monocotyledons and dicotyledons. We found that the intensity of CG independent selection correlates positively and of TA independent selection correlates negatively with the levels of genome evolution. Of the leftmost five green algae genomes in Fig. 3b, *Bathycoccus prasinos*, *Coccomyxa subellipsoidea*, *Eudorina*, *Monoraphidium* and *Picochlorum*, their TA independent selection are obviously high and their CG inhibition are strongly. We thought that the five green algae genomes are evolving very slowly or they are ancient species.

#### Fungus genomes

We found that the phenomenon of the mutual inhibition and the strong mutual inhibition also exists in fungus genomes. The intensity distribution pattern of *Agaricomycotina* and *Pezizomycotina* genomes is similar, and they are different from the distribution pattern of *Saccharomycetales* genomes (Fig. 3c–e). In *Agaricomycotina* and *Pezizomycotina* genomes, the TA independent selection is very obvious and the strong CG inhibition is present, but there is no strong TA inhibition. In the *Saccharomycetales* genomes, the CG independent selection is very obvious and the strong TA inhibition is present, but there is no strong CG inhibition. The independent selection pattern of *Saccharomycetales* genomes is similar to that of invertebrate genomes.

We found that the independent selection pattern of species genomes is closely related to the living habits of the species. For *Agaricomycotina* genomes (Fig. 3c) with remarkable TA independent selection and strong CG inhibition, such as *Trametes versicolor* and *Trametes pubescens*, they usually grow on trees, and with remarkable CG independent selection, such as *Termitomyces* and *Leucoagaricus*, they usually live in the soil. For *Pezizomycotina* genomes (Fig. 3d) with remarkable TA independent selection and strong CG inhibition, such as *Purpureocillium lilacinum* and *Tolypocladium phioglossoides*, they mainly infect plants, and with remarkable CG independent selection, such as *Blastomyces gilchristii* and *Blastomyces dermatitidis*, they mainly infect animals, such as human. In *Saccharomycetales* genomes, the genome with remarkable CG independent selection and strong TA inhibition is *Banseniaspora guilliermondii* (Fig. 3e).

#### Prokaryote genomes

We found that the mutual inhibition and the strong mutual inhibition relationships exist obviously in prokaryote genomes. The independent selection pattern of species genomes is closely related to the living habits of the species.

In archaea genome group, the genomes with remarkable TA independent selection and strong CG inhibition are *Halobacteria* and *Methanomicrobia* genomes. Conversely, the genomes with remarkable CG independent selection and strong TA inhibition are *Methanomada* and *Thermoprotei* genomes (Fig. 3f). For *Halobacteria* genomes with the most remarkable TA independent selection and strong CG inhibition, such as *Halosimplex *sp. TH32, *Halarchaeum acidiphilum* MH1-52-1 and *Halarchaeum *sp. CBA1220 (The leftmost genomes in Fig. 3f), they are the halophilic bacteria. For the *Methanomada* genomes with remarkable CG independent selection and strong TA inhibition, some of them have been found in deep-sea hydrothermal vents or marsh gas environments, such as *Methanocaldococcus jannaschii* and *Methanobrevibacter arboriphilus* ANOR1, some of them have been found in the stomachs, the intestines and gingiva of animals, such as *Methanosphaera cuniculi*, *Methanobrevibacter olleyae* and *Methanobrevibacter oralis* (The rightmost genomes in Fig. 3f).

In eubacteria genomes, most of *Actinobacteria* and part of *Proteobacteria* genomes have remarkable TA independent selection and strong CG inhibition, and most of *Spirochaetales* genomes and part of *Firmicutes* genomes have remarkable CG independent selection and strong TA inhibition (Fig. 3g). For *Actinobacteria* genomes with the most remarkable TA independent selection and strong CG inhibition, such as *Agrococcus sp*. SGAir0287, *Agrococcus jejuensis* and *Agrococcus carbonis* (the leftmost three genomes in Fig. 3g), they live in soil and water and usually infect plants and fungi. For *Spirochaetales* genomes with the most remarkable CG independent selection and strong TA inhibition, such as *Borrelia recurrentis*, *Borrelia duttonii* Ly and *Borrelia miyamotoi* LB-2001 (the rightmost three genomes in Fig. 3g), they live in soil and decaying organic matter and usually infect animals, such as human.

Based on our results, we proposed an evolution mechanism of genomes in which genome evolution is determined by the intensities of CG and TA independent selections and the mutual inhibition relationship between CG and TA independent selections.

### Independent selection laws and G + C content of genome sequences

**Fig. 4:**
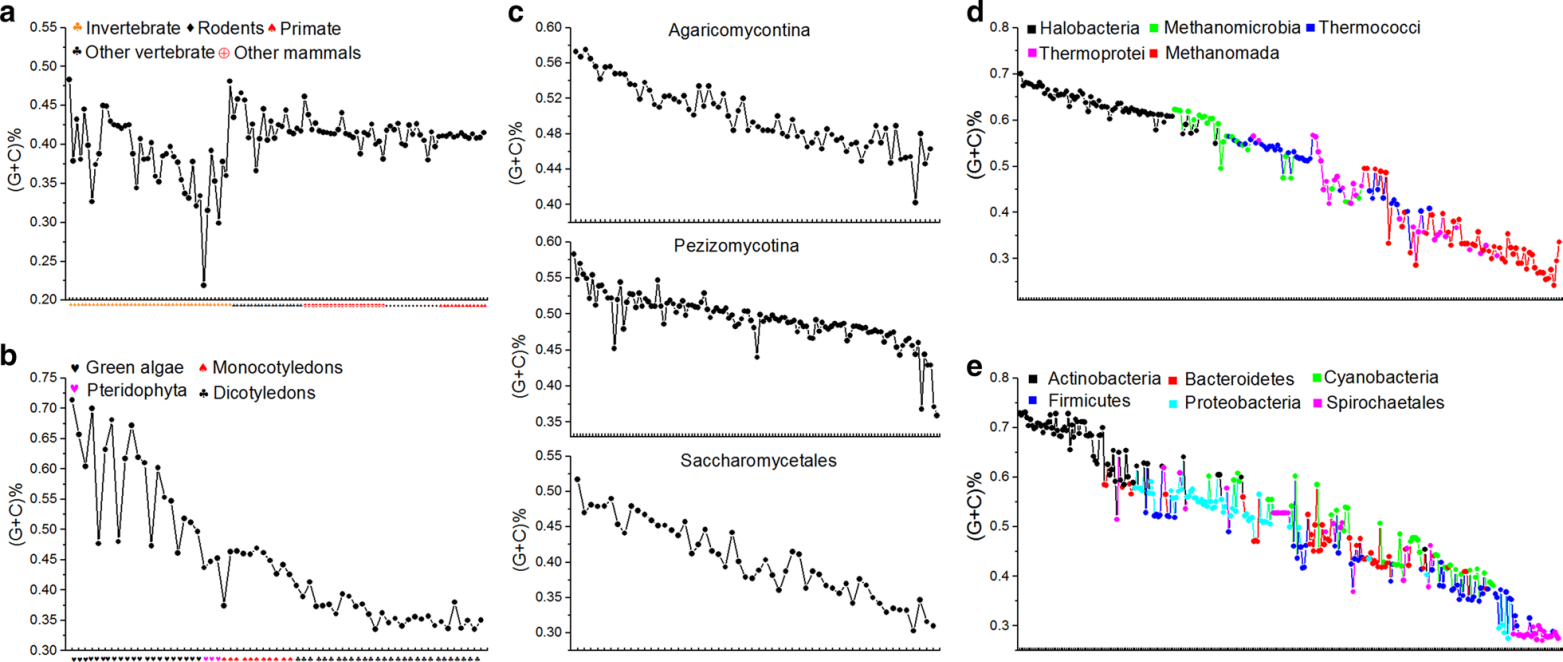
Relations between the G + C content of genome sequences and the independent selections. The abscissa in each figure is the species genomes which are arranged as the same as in Fig. 3. The species in each species group are arranged by its separability value of CG1 motif spectrum from small to large. **a** Animal genomes. From left to right, the species group is invertebrate, other vertebrate, other mammals, rodents and primates. **b** Plant genomes. From left to right, the species group is green algae, pteridophyte, monocotyledons and dicotyledons. **c** Fungus genomes. The figure above is *Agaricomycotina* genomes, the figure in the middle is *Pezizomycotina* genomes and the following figure is *Saccharomycetales* genomes. **d** Archaea genomes. They are classified into five subgroups: *Halobacteria* (black), *Methanomicrobia* (green), *Thermococci* (blue), *Thermoprotei* (pink), and *Methanomada* (red). **e** Eubacteria genomes. They are classified into six subgroups: *Actinobacteria* (black), *Proteobacteria* (wathet blue), *Cyanobacteria* (green), *Bacteroidetes* (red), *Firmicutes* (blue) and *Spirochaetales* (pink)

G + C content is the most basic characteristic quantity to describe the composition of DNA sequences. Here, we analyzed the relations between the two independent selection laws and G + C content of genome sequences. Besides primate and rodent genomes, we found that ${\delta }_{CG1}$/${\delta }_{CG2}$ and ${\rho }_{CG1}$/${\rho }_{CG2}$ correlate negatively and significantly with G + C content of genome sequences, ${\delta }_{TA1}$/${\delta }_{TA2}$ and ${\rho }_{TA1}$/${\rho }_{TA2}$ correlate positively and significantly with G + C content of genome sequences. It indicates that the intensities of CG independent selection correlates negatively and of TA independent selection correlates positively with G + C content of genome sequences. In primate and rodent genomes, there are not obvious correlations between CG independent selection and G + C content, and there are not consistent correlations between TA independent selection and G + C content (Additional file 4: Table S3 and Fig. 4). We thought that disappeared mutual inhibition relationship in primate and rodent genomes is the main reason to bring about the correlations weakened or disappeared.

We can explain the correlation between the independent selections and the G + C content by the mechanism of genome evolution. In theory, the average G + C content is 65.34% in CG1 6-mer subset, 43.75% in CG0 6-mer subset, 34.65% in TA1 6-mer subset and 56.24% in TA0 6-mer subset. When ${\delta }_{CG1}$ goes up, because the mutual inhibition between the CG and TA independent selections, ${\delta}_{TA1}$ must go down. That is to say ${\stackrel{-}{x}}_{CG1}$ goes down or the total number of 6-mers appeared in CG1 6-mer spectrum decrease and ${\stackrel{-}{x}}_{TA1}$ goes up or the total number of 6-mers appeared in TA1 6-mer spectrum must increase (Additional file 5: Figure S1A, B and Additional file 3: Animation S1). The number of CG1 6-mers with high G + C content decrease and the number of TA1 6-mer with low G + C content increase, the two situations lead to a decrease of G + C content in genome sequence. Conversely, when ${\delta }_{CG1}$ goes down, ${\delta}_{TA1}$ must go up. The two kinds of situations must lead to an increase of G + C content in the genome sequence (Additional file 5: Figure S1C, D and Additional file 3: Animation S1). Thus, the deeper biological significance of G + C content was revealed through the mechanism of genome evolution. We concluded that G + C content of genome sequences is a comprehensive representation of genome evolution.

### Evolution states and process of prokaryote genomes in the beginning of life

The intensity distributions of the two independent selections for 920 genomes (Fig. 3) showed us abundant images which represent not only the evolution state of genomes at present but also the evolutionary states and evolutionary process of genomes at the early stages of life. Here, we used the idea in astronomy to speculate the process of genome evolution in the early stages of life. In astronomy, the evolution of stars can be obtained by studying the sky photographs at present. Countless stars show us not only the state of stars at “present” but also the evolution process of stars, which can be derived by a variety of stars with different evolving states. In the maps of the intensity distribution, when the abscissa is considered a variety of species genomes, the distribution represents the evolution state of different species at present. When the abscissa is considered a timeline of one species genome, the distribution represents the evolution process of the species from ancient to now.

Our results indicate that higher animals or higher plants usually have obvious CG independent selection and lower animals or lower plants usually have obvious TA independent selection. It is known that prokaryotes first originated in oceans and lakes and there was no oxygen in them and the earth's atmosphere. It is called anaerobic environment. If the intensity distribution of eubacteria genomes (Fig. 3g) is considered as a timeline of one species genome, we could conclude that the TA independent selection is the dominated mode of prokaryote genomes in the anaerobic environment. The TA independent selection mode was suitable for prokaryotes to live in the anaerobic environment.

The intensity distribution of archaea genomes showed us another images. Although the TA independent selection is the main evolution mode of early prokaryotes under the anaerobic environment on the earth, in order to live around the extreme environment, such as living in the deep-sea hydrothermal vents or marsh gas, some of the prokaryotes were changing gradually the evolution mode from TA independent selection to CG independent selection to adapt to the extreme environment, but the TA independent selection must be inhibited. In order to live in salt water (another kind of extreme environment), some of the prokaryotes still insisted on the evolution mode of TA independent selection, but the CG independent selection must be inhibited (Fig. 3f and “Prokaryote genomes” section). We can see that the mechanism of mutual inhibition between CG and TA independent selections is a nature selected way for prokaryotes living in the extreme environments. Thus, there were three kinds of prokaryotes in that time. The first one was the prokaryotes with obvious CG independent selection and TA inhibition, and the second one was the prokaryote with obvious TA independent selection and CG inhibition. They all lived in extreme environments. The two kinds of prokaryotes are called archaea bacteria. The third one was the prokaryote with obvious TA independent selection, but they lived in a relatively mild environment and they are called eubacteria. Archaea has stronger environmental adaptability and evolutionary ability than eubacteria.

As early prokaryotes gradually released oxygen, the concentration of oxygen in the earth's oceans and lakes gradually increased, and so did the concentration of oxygen in the atmosphere. The aerobic environment is another extreme environment. From the anaerobic environment to the aerobic environment, all of the prokaryotes had to adapt to the aerobic environment. For eubacteria, the TA independent selection mode could not adapt to the oxygen increasing in a short time, due to the weak adaptability, most of them died out and only few of them survived. This is the great oxidation event 2 billion years ago. When standing in the perspective of the timeline to observe the intensity distribution of eubacteria (Fig. 3g), it is found that the survived eubacteria had to adopt the two different strategies to live in the aerobic environment. The one is to enhance the intensity of TA independent selection, but the intensity of CG independent selection must be inhibited. The other strategy is to try to transform the mode of TA independent selection into the mode of CG independent selection and the TA independent selection must be inhibited. We can see that the mechanism of mutual inhibition between CG and TA independent selections is a nature selected way for eubacteria living in the aerobic environments. For archaea with obvious CG independent selection and TA inhibition, the aerobic environment was exactly a suitable environment, this condition prompted the archaea to leave the extreme environment and live anywhere in the aerobic environment. For the archaea with obvious TA independent selection and CG inhibition, this evolution pattern was also suitable for the archaea living in aerobic environment. Then, this kind of archaea could also leave the salt environment and live anywhere in the aerobic environment (Fig. 3f). We considered that the strong adaptability and evolutionary ability of archaea genomes and the stimulation of the aerobic environment are the main reasons to lead to the species transformation from prokaryote to eukaryote.

### Early evolution of eukaryote genomes

When standing in the perspective of the timeline and comparing the intensity distributions of eukaryote genome groups in the early stages of life (left parts in Fig. 3), we found that there are two different distribution patterns. The one is it happens in animals and *Saccharomycetales* and their intensity distributions are similar. The common features are that the CG independent selection is obvious and the TA independent selection is inhibited (see the right part of Fig. 3a, e). The other is it happens in plants, *Agaricomycotinas* and *Pezizomycotinas* and their intensity distributions are similar. The common features are that the TA independent selection is obvious and the CG independent selection is inhibited (see the left part of Fig. 3b–d). Carl Woese’s Three Domain theory pointed out those eukaryotes originated from archaea and not from eubacteria [22]. We found that the evolution pattern of the archaea genomes with obvious CG independent selection and TA inhibition is similar to that of animals and *Saccharomycetales*, the evolution pattern of the archaea with obvious TA independent selection and CG inhibition is similar to that of plants, *Agaricomycotinas* and *Pezizomycotinas*. Based on the continuity and the similarity of genome evolution modes, we considered that the ancestor of animals and *Saccharomycetales* originated from the archaea with obvious CG independent selection and TA inhibition, and the ancestor of plants, *Agaricomycotinas* and *Pezizomycotinas* originated from the archaea with obvious TA independent selection and CG inhibition. The origin and early evolution of different eukaryotic lineages may have different selection modes at genome level.

### The independent selection modes and the living habits of species

We found that the independent selection mode adopted by species genomes is closely related to the living habits of the species (see ‘Prokaryote genomes’ sections). The eubacteria with obvious TA independent selection and strong CG inhibition, such as Actinobacteria, usually live with or infect the eukaryote species which originated from the archaea with obvious TA independent selection and CG inhibition, such as plants. The eubacteria and archaea with obvious CG independent selection and strong TA inhibition, such as *Spirochaetales* and *Methanomada*, usually live with or infect the eukaryote species which originated from the archaea with obvious CG independent selection and TA inhibition, such as animals. That means similar evolution modes of genomes determine the interaction preference between prokaryotes and eukaryotes.

## Discussion

The independent selection modes of genome sequence are closely related to the G + C content of genome sequence. Therefore, the G + C content is an expression of sequence evolution rules. It is known that G + C content is not uniform and always clusters in DNA sequences. By analyzing the G + C content distribution in DNA sequences, we can reveal the evolution states of local DNA segments or the evolution states of different sequences, such as protein-coding sequences, introns and CpG island sequences, etc. It is helpful for us to understand deeply the origination and evolution relations of different sequences.

Genome evolution is a continuous process. Although the phenomenon of TA independent selection has disappeared basically in primate and rodent genomes, the trace of TA independent selection still exists and the corresponding functional elements are still reserved. For example, due to the commonness of coding rules in protein-coding sequences, the trace of TA independent selection must be inherited in protein-coding sequences of human and rodent genomes. To verify this, the spectra of three TA and three CG motif subsets of protein-coding sequences in human and mouse genomes were presented in Additional file 5: Figure S2. Although the CG independent selection is obvious, it can be seen that the TA independent selection is also obvious in protein-coding sequences of human and rodent genomes.

Based on the evolution mechanism of genomes, it is possible to solve the puzzles encountered in studying the evolutionary relationships of genomes. K-mer frequencies of genome sequence include the information of whole genome sequence at the level of sequence composition. When we use the total k-mer frequencies to study the genome evolution relations, it avoids the disadvantage of using partial sequence information instead of whole genome sequence information. Because some researchers did not know what kinds of k-mers are sensitive to genome evolution, they had to filter out some k-mers in total k-mer set to obtain the acceptable phylogenetic trees, such as filtering the k-mers with the highest or lowest frequencies. Filtering out some k-mers destroys the integrity of genomic information. Since the selected k-mer number has no theoretical support and has a certain degree of arbitrariness, it cannot obtain a consistent evolutionary relationship of species, and it cannot be used as a standard for species identification. Independent selection laws show that there are three types of independent k-mers and the spectra of the k-mers containing CG or TA dinucleotide are sensitive to genome evolution and the spectra of CG0/TA0 k-mers reflect the basic structures of a genome sequence. Thus, the three types of k-mers contribute differently to genome evolution. If we can consider the weighting factors of the three types of k-mers and do not filter any k-mer, we thought it is the most reasonable method to construct the evolutionary relationship among species genomes.

Our results have important guiding significance for biological information mining of nucleotide sequences. The independent selection laws reveal the composition rule of nucleotide sequences. It shows that the three kinds of CG motifs and the three kinds of TA motifs have evolutionary independence, and the k-mers containing CG and TA dinucleotides are functional motifs. That is to say, any nucleotide sequence is composed by the six kinds of motifs. The proportion of these motifs and their distribution forms in a nucleotide sequence determine its biological functions. If biological information mining in nucleotide sequences is considered in this way, the problem will become clear and simple. Our proposal may provide us with a new idea from theory to sequence.

Our results showed that the living habits of species are closely related to the independent selection mode adopted by species genomes. We can study further the interaction relationships between different species from the perspective of the independent selections of genome sequences. Such as, why some bacteria infect plants and why some others only infect animals.

The CG and TA independent selection laws and their mutual inhibition relationships in genome sequences have been revealed by studying the intrinsic laws of k-mer spectra of genome sequences. But the relations between the sequence structure of each k-mer and its occurrence frequency in genome sequence are not clear. Just as the atomic structure was revealed by studying the laws of atomic spectra, we believe that the mechanism of the composition and the evolution of genome sequences will be improved further by studying the structures and usage of all k-mers in genome sequences.

## Conclusions

We revealed the intrinsic laws of various motif spectra of genome sequences based on 920 genome sequences from human to bacteria, and found that there are two kinds of evolution selection modes in genome sequences. One is named as the CG independent selection law and the TA independent selection law. The two independent selection laws have five properties: evolution independence, evolution selectivity, evolution conservatism, evolution correlation and evolution homoplasy. We found that there is the mutual inhibition relationship between the CG independent selection and TA independent selection in species genomes and proposed an evolution mechanism of genomes in which the genome evolution is determined by the intensities of the CG and TA independent selections and the mutual inhibition relationship. The intensity of the CG independent selection correlates positively and of TA independent selection correlates negatively with the levels of genome evolution in animals and plants. The intensity of TA independent selection correlates positively and of the CG independent selection correlates negatively with the G + C content of genome sequences. Besides, by the evolution mechanism of genomes, we speculated the evolution modes of prokaryotes in mild and extreme environment in the anaerobic age and the evolving process of prokaryotes from the anaerobic environment to the aerobic environment. Based on the continuity and similarity of genome evolving modes, we speculated that the ancestor of animals and *Saccharomycetales* originated from the archaea with obvious CG independent selection and TA inhibition, and the ancestor of plants, *Agaricomycotinas* and *Pezizomycotinas* originated from the archaea with obvious TA independent selection and CG inhibition. The independent selection mode adopted by species genomes is closely related to the living habits of the species.

## Methods

### Genome data

Genome sequences and the corresponding annotation information come from UCSC (http://genome.ucsc.edu/) and Gene Bank (http://www.ncbi.nlm.nih.dov/genbank). The 920 genomes included 69 vertebrates, 43 invertebrates, 63 plants, 245 fungi, 200 archaea and 300 eubacteria. X chromosomes were not selected in each genome. The species genome taxonomy and the abbreviation of each species group were shown in Table 1, and the detailed information was given in Additional file 1: Table S1.

### K-mer spectrum

For a given DNA sequence with length *L* bp, the occurrence frequencies of each k-mer is calculated by taking k bp as the window and 1 bp as the sliding step. If the number is *N*_*i*_ for the k-mers with frequency *i*, the relative motif number (*RMN*) in the frequency block *i* is defined as the following:

1 $$RMN=\frac{{N}_{i}}{{4}^{k}}$$

Taking k-mer frequency as the abscissa and the relative motif number as the ordinate, the distribution of relative motif number with k-mer frequency is obtained, which is called k-mer frequency spectrum or k-mer spectrum.

### XY dinucleotide classification

In order to analyze the relations between the occurrence frequencies and the composition characteristics of k-mers, all k-mers are classified into a series of k-mer subsets. We proposed a classification method called XY dinucleotide classification. The method is defined as following:

For 8-mers, if the 8-mer does not contain XY dinucleotide (Here X and Y stand for A, C, G or T), it is called XY0 8-mers, contains one XY dinucleotide, called XY1 8-mers and contains two or more than two XY dinucleotide, called XY2 8-mers. Thus, there are 16 classification methods, and all 8-mers in each classification is divided into three 8-mer subsets XY0, XY1 and XY2. Theoretically, when X and Y are not the same, the number of XY0 8-mer subset is 40,545, of XY1 8-mer subset is 21,468 and of XY2 8-mer subset is 3523. When X and Y are the same, the number of XY0 8-mer subset is 44,631, of XY1 8-mer subset is 14,931 and of XY2 8-mer subset is 5974.

For 6-mers, if the 6-mer does not contain XY dinucleotide, it is called XY0 6-mers and contains one or more than one XY dinucleotide, called XY1 6-mers. Theoretically, when X and Y are not the same, the number of XY0 6-mer subset is 2911 and the number of XY1 6-mer subset is 1185. When X and Y are the same, the number of XY0 6-mer subset is 3105 and the number of XY1 6-mer subset is 991. Thus, there are 16 classification methods, and all 6-mer in each classification is divided into two motif subsets XY0 and XY1.

Then, we can obtain the spectrum distributions for all of the 8-mer (or the 6-mer) subsets.

### The average frequency and the standard deviation of k-mer spectrum

For a given k-mer spectrum with normal distribution, the average frequency and the standard deviation are used to describe the spectrum features. Their definitions are as following:

1. The average frequency of a spectrum
  2 $$\stackrel{-}{x}=\frac{\sum {x}_{i}}{N}$$
2. The standard deviation of a spectrum
  3 $$SD=\sqrt{\frac{\sum {({x}_{i}-\stackrel{-}{x})}^{2}}{N-1}}$$

Here, *x*_*i*_ is the frequency of the *i*th k-mer in a given k-mer subset, *N* is the total number of the k-mers in the k-mer subset.

### Separability and conservatism

In order to compare the location differences and the conservatism (just like the monochromaticity in optics) of a spectrum of given k-mer subset with that of the spectrum of total k-mers of a genome sequence, the separability and the conservatism are defined as following:

1. Separability of a spectrum
  4 $${\delta }_{i}=\frac{\stackrel{-}{x}}{{\stackrel{-}{x}}_{i}}$$
2. Conservatism of a spectrum
  5 $${\rho }_{i}=\frac{SD}{{SD}_{i}}$$

Here, *SD* is the standard deviation of the spectrum distribution of total k-mers and *SD*_*i*_ is the standard deviation of the spectrum distribution of the *i*th k-mer subset. The ${\rho }_{i}$ represents the relative value of the conservatism for the spectrum of *i*th subset compared with the spectrum of total motifs. The larger ${\rho }_{i}$ is, the more conservative the spectrum distribution of the *i*th k-mer subset, that is to say, the more concentrated the frequency distribution of the k-mer subset.

The two characteristic quantities have nothing to do with the number of k-mer subsets and the absolute position (k-mer frequencies) of k-mer spectra. Thus, we can use the two quantities to compare the differences of the spectra of any k-mer subsets in a genome or among different genomes.

## Supplementary information

**Additional file 1: Table S1.** The values of the separability and the conservatism for 920 genomes.

**Additional file 2: Table S2.** The point estimation of the separability and the conservatism for all XY 8-mer subsets in each species group.

**Additional file 3: Animation S1.** The mutual inhibition relationship between CG and TA independent selections.

**Additional file 4: Table S3.** Linear correlation coefficients between the G+C content and the intensities of CG and TA independent selections in each species group.

**Additional file 5: Figure S1.** The spectrum distributions of CG1 and TA1 6-mer subsets. The vertical broken line (middle) represents the average 6-mer frequency of the corresponding genome sequence. (A) *Methanocaldococcus infernus* genome (archaea) that has remarkable CG independent selection and strong TA inhibition. (B) *Borrelia recurrentis* A1 genome (eubacteria) that has remarkable CG independent selection and strong TA inhibition. (C) *Halosimplex* genome (archaea) that has remarkable TA independent selection and strong CG inhibition. (D) *Agrococcus* sp. SGAir0287 genome (eubacteria) that has remarkable TA independent selection and strong CG inhibition. **Figure S2.** The 8-mer spectrum distributions. (**A**) TA2, TA1 and TA0 8-mer spectra of protein coding sequences in human genome. (**B**) CG2, CG1 and CG0 8-mer spectra of protein coding sequences in human genome. (**C**) TA2, TA1 and TA0 8-mer spectra of protein coding sequences in mouse genome. (**D**) CG2, CG1 and CG0 8-mer spectra of protein coding sequences in mouse genome.

## Data Availability

All 920 genome sequences and the corresponding annotation information were obtained from GenBank (http://www.ncbi.nlm.nih.dov/genbank) and UCSC (http://genome.ucsc.edu/). X chromosomes were not selected in each genome. Supporting material are publicly available at http://120.53.4.254/Kmerspectra/data.php.

## Abbreviations

RMN: Relative motif number
XY0: The 8-mers or 6-mers contain none XY dinucleotide
XY1: The 8-mers contain one XY dinucleotide or the 6-mers contain more than one XY dinucleotide
XY2: The 8-mers contain more than two XY dinucleotide
