# Supplementary material for: Intrinsic laws of k-mer spectra of genome sequences and evolution mechanism of genomes
Source: BMC Evol Biol. 2020 Nov 23;20:157. doi: 10.1186/s12862-020-01723-3 (PMC7684957; doi:10.1186/s12862-020-01723-3)
Supplement: Supplementary file 4 — Additional file 4: Table S3. Linear correlation coefficients between the G+C content and the intensities of CG and TA independent selections in each species group. [file 12862_2020_1723_MOESM4_ESM.docx]

**Additional Table S3** Linear correlation coefficients between the G+C content and the intensities of CG and TA independent selections in each species group

|  |  |  | Animal |  |  | Plant | Fungus | Archaca | Eubacteria |
| --- | --- | --- | --- | --- | --- | --- | --- | --- | --- |
|  | Pri | Rod | Mam | Vrt | Inv |  |  |  |  |
|  | (13) | (14) | (22) | (20) | (43) | (63) | (245) | (200) | (300) |
| δ_CG2_-G+C% | 0.210 | -0.144 | -0.675^**^ | -0.482^*^ | -0.524^**^ | -0.860^**^ | -0.812^**^ |  |  |
| δ_CG1_-G+C% | 0.221 | -0.516 | -0.697^**^ | -0.472^*^ | -0.601^**^ | -0.895^**^ | -0.875^**^ | -0.897^**^ | -0.886^**^ |
| ρ_CG2_-G+C% | 0.248 | -0.167 | -0.576^**^ | -0.656^**^ | -0.737^**^ | -0.740^**^ | -0.808^**^ |  |  |
| ρ_CG1_-G+C% | 0.385 | -0.342 | -0.702^**^ | -0.550^*^ | -0.674^**^ | -0.844^**^ | -0.794^**^ | -0.758^**^ | -0.721^**^ |
| δ_TA2_-G+C% | 0.669^*^ | 0.749^**^ | 0.767^**^ | 0.739^**^ | 0.706^**^ | 0.916^**^ | 0.785^**^ |  |  |
| δ_TA1_-G+C% | -0.227 | 0.743^**^ | 0.853^**^ | 0.760^**^ | 0.816^**^ | 0.948^**^ | 0.925^**^ | 0.956^**^ | 0.903^**^ |
| ρ_TA2_-G+C% | 0.568^*^ | 0.400 | -0.384 | 0.148 | 0.273 | 0.833^**^ | 0.348^**^ |  |  |
| ρ_TA1_-G+C% | 0.514 | 0.638^*^ | 0.368 | 0.472^*^ | 0.426^**^ | 0.891^**^ | 0.715^**^ | 0.929^**^ | 0.768^**^ |

Note:Two-tailde significance:^*^,P<0.05;^**^,P<0.01.
