# Supplementary material for: Intrinsic laws of k-mer spectra of genome sequences and evolution mechanism of genomes
Source: BMC Evol Biol. 2020 Nov 23;20:157. doi: 10.1186/s12862-020-01723-3 (PMC7684957; doi:10.1186/s12862-020-01723-3)
Supplement: Supplementary file 5 — Additional file 5: Figure S1. The spectrum distributions of CG1 and TA1 6-mer subsets. The vertical broken line (middle) represents the average 6-mer frequency of the corresponding genome sequence. (A) Methanocaldococcus infernus genome (archaea) that has remarkable CG independent selection and strong TA inhibition. (B) Borrelia recurrentis A1 genome (eubacteria) that has remarkable CG independent selection and strong TA inhibition. (C) Halosimplex genome (archaea) that has remarkable TA independent selection and strong CG inhibition. (D) Agrococcus sp. SGAir0287 genome (eubacteria) that has remarkable TA independent selection and strong CG inhibition. Figure S2. The 8-mer spectrum distributions. (A) TA2, TA1 and TA0 8-mer spectra of protein coding sequences in human genome. (B) CG2, CG1 and CG0 8-mer spectra of protein coding sequences in human genome. (C) TA2, TA1 and TA0 8-mer spectra of protein coding sequences in mouse genome. (D) CG2, CG1 and CG0 8-mer spectra of protein coding sequences in mouse genome. [file 12862_2020_1723_MOESM5_ESM.docx]

**Figure S1**

**
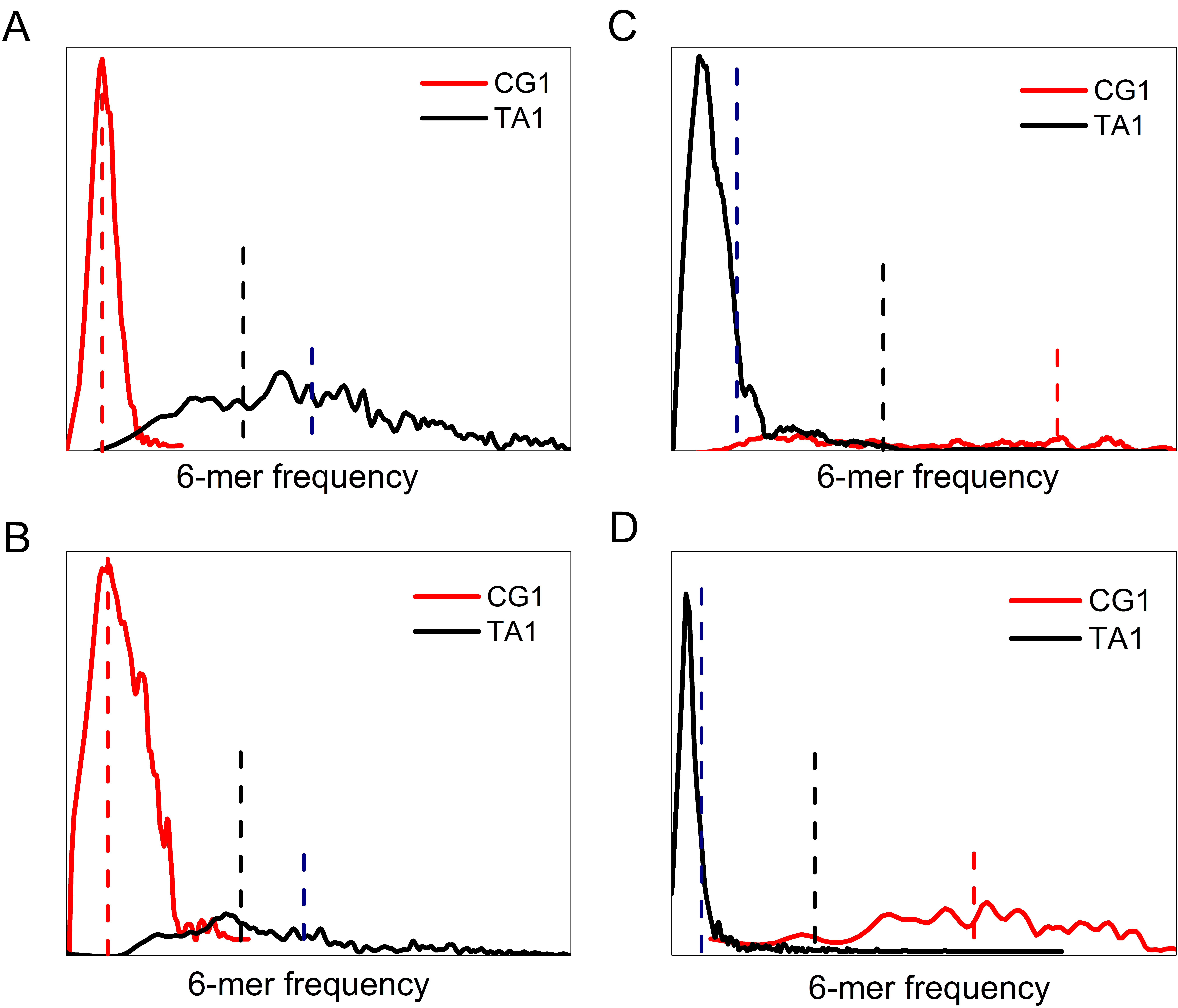
**

**Figure S2**


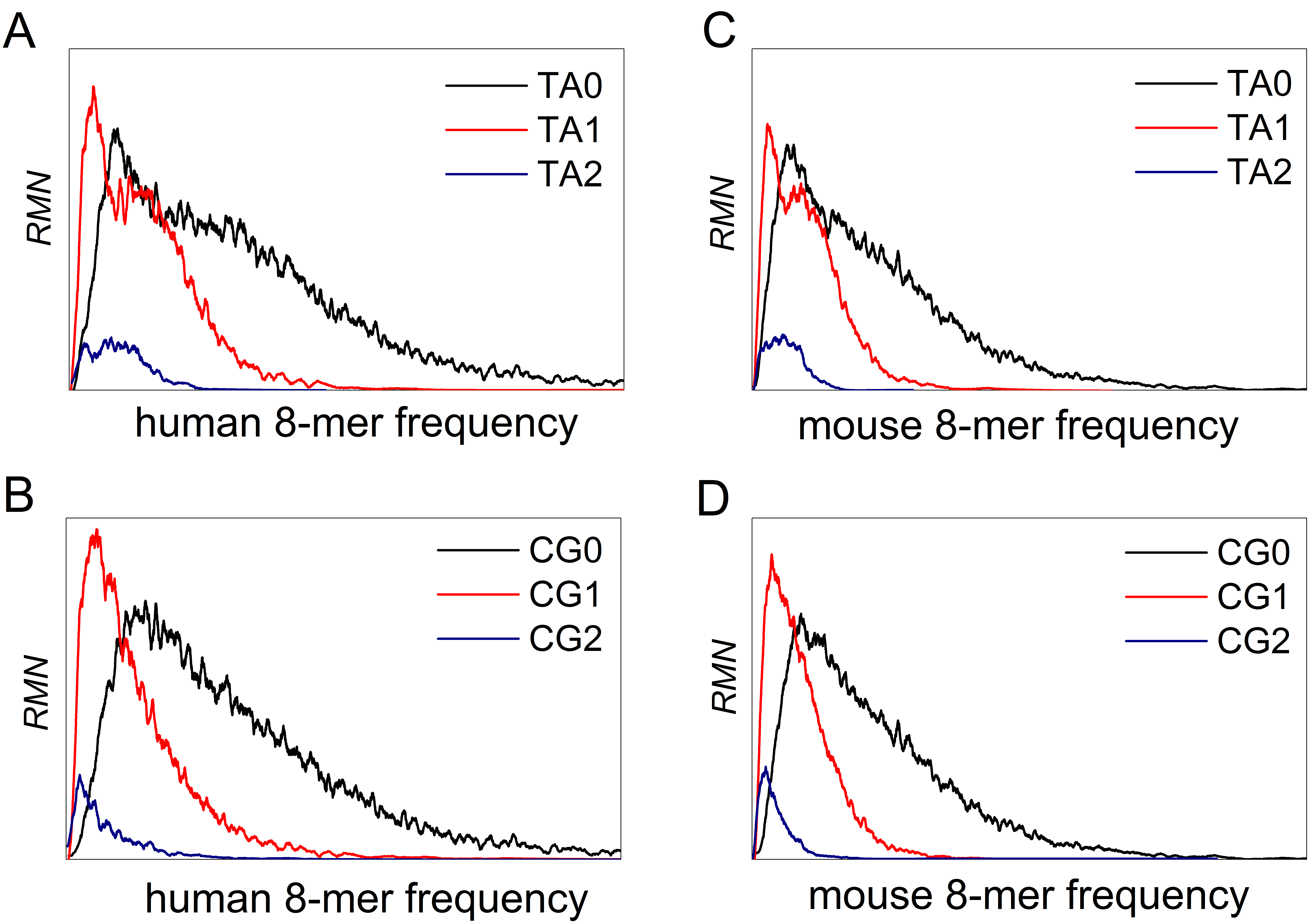


Additional Figure Legends

**Figure S1** The spectrum distributions of CG1 and TA1 6-mer subsets. The vertical broken line (middle) represents the average 6-mer frequency of the corresponding genome sequence. (A) *Methanocaldococcus infernus* genome (archaea) that has remarkable CG independent selection and strong TA inhibition. (B) *Borrelia recurrentis* A1 genome (eubacteria) that has remarkable CG independent selection and strong TA inhibition. (C) *Halosimplex* genome (archaea) that has remarkable TA independent selection and strong CG inhibition. (D) *Agrococcus sp*. SGAir0287 genome (eubacteria) that has remarkable TA independent selection and strong CG inhibition.

**Figure S2** The 8-mer spectrum distributions. (**A**) TA2, TA1 and TA0 8-mer spectra of protein coding sequences in human genome. (**B**) CG2, CG1 and CG0 8-mer spectra of protein coding sequences in human genome**.** (**C**) TA2, TA1 and TA0 8-mer spectra of protein coding sequences in mouse genome. (**D**) CG2, CG1 and CG0 8-mer spectra of protein coding sequences in mouse genome.
